# The influence of Coulomb interaction screening on the excitons in disordered two-dimensional insulators

**DOI:** 10.1038/s41598-021-91414-w

**Published:** 2021-06-07

**Authors:** E. V. Kirichenko, V. A. Stephanovich

**Affiliations:** Institute of Physics, Opole University, Oleska 48, 45-052 Opole, Poland

**Keywords:** Two-dimensional materials, Quantum mechanics, Theoretical physics

## Abstract

We study the joint effect of disorder and Coulomb interaction screening on the exciton spectra in two-dimensional (2D) structures. These can be van der Waals structures or heterostructures of organic (polymeric) semiconductors as well as inorganic substances like transition metal dichalcogenides. We consider 2D screened hydrogenic problem with Rytova–Keldysh interaction by means of so-called fractional Scrödinger equation. Our main finding is that above synergy between screening and disorder either destroys the exciton (strong screening) or promote the creation of a bound state, leading to its collapse in the extreme case. Our second finding is energy levels crossing, i.e. the degeneracy (with respect to index $\mu$) of the exciton eigenenergies at certain discrete value of screening radius. Latter effects may also be related to the quantum manifestations of chaotic exciton behavior in above 2D semiconductor structures. Hence, they should be considered in device applications, where the interplay between dielectric screening and disorder is important.

## Introduction

In the last decade, the quest for ultra-thin, low-cost photovoltaic cells, light-emitting diodes, and other electronic devices^1,2^ has stimulated much research on amorphous and disordered semiconductors^3–10^. While significant progress has been made in the understanding many features of exciton diffusion and dissociation in them, important questions remain pertaining to the fundamental physics underlying devices functionality, especially for the geometrically confined structures like surfaces, interfaces, and quantum wells. For example, the role of Coulomb interaction and its screening in two-dimensional systems needs to be addressed^11^. This is especially true for semiconductors, where the dielectric screening is particularly strong. The disordered semiconductors envisioned for photovoltaic applications, are primarily polymers^3,10,12–14^. There are different kinds of disorder in these materials—even if the molecular composition of a sample is well controlled, there are still many kinds of unavoidable imperfections (like conformational and structural disorder, presence of chemical impurities, etc, see, e.g.^15^), which adversely influence the functionality of a corresponding electronic device.

To describe the above disorder, one usually invokes Gaussian distributions, which are sufficient for a not high concentration of disordered constituents. This is because such case corresponds to a weak (or absence of) disorder, where the width of the distribution function is always small, corresponding to an “almost deterministic” situation. If the concentration of defects and/or impurities is large, the randomness becomes high so that the width of the corresponding distribution elevates. In such case, the Gaussian approximation is often insufficient (see also below, "Disorder and heavy-tailed Lévy distributions" section) so that more general, Lévy-type distributions should be utilized^16^. The random elementary trajectories of Lévy processes consist of continuous motions interspersed with long excursions, which are “responsible” for the so-called long tails of such distributions, see, e.g.^17^ and references therein. Under long tails we mean that Lévy distributions usually have power-law decay $x^{-1-\mu }$ ($0<\mu <2$ is so-called Lévy index), i.e. much slower than Gaussian^17^. As the decay law of probability density function determines its width, we can see that the Lévy index $\mu$ is actually responsible for the width of the corresponding distribution. We note here, that at $\mu =2$ the Lévy distribution coincides with Gaussian^16,17^, thus corresponding to weak disorder case. Since the width is directly proportional to the “degree of disorder” (i.e. “how random” our system is), we can assert, that the Lévy index $\mu$ determines that degree. In other words, the “more random” is our system, the more $\mu$ deviates from 2 (Gaussian case). The above weak decay implies that Lévy distributions have divergent variance, which is the subject of the generalized central limit theorem, proved by Lévy^18^. It turns out that Lévy distributions occur in many physical^19–22^, biological^22,23^, and financial^24,25^ systems. Technically, latter non-Gaussian distributions appear in the solutions of so-called pseudo-differential equations with fractional derivatives^22,23^, see below for mathematical details.

One more interesting application of Lévy processes is so-called fractional quantum mechanics^26^, dealing in short with the substitution of the ordinary Laplacian with the fractional one in the stationary Schrödinger equation. The solution to such a problem, if it exists, represents the spectrum of a quantum system, subjected to the above strong disorder.

In the present paper, we account for disorder in 2D hydrogenic problem by substitution of the ordinary Laplacian by its fractional analog of order $\mu$, in the corresponding Scrödinger equation^26^. This implies, that we consider the disorder phenomenologically substituting the conventional Laplacian by the fractional one in the Schrödinger equation, describing the structure of an exciton.

Our main motivation is that while the influence of disorder on the excitons motion (like diffusion, which may become sub- or superdiffusion in disordered substances) is rather well studied, there is no model, describing the influence of disorder on the exciton spectrum by itself. The detailed discussion of this supposition is presented in the next "Disorder and heavy-tailed Lévy distributions" section.

**Figure 1:**
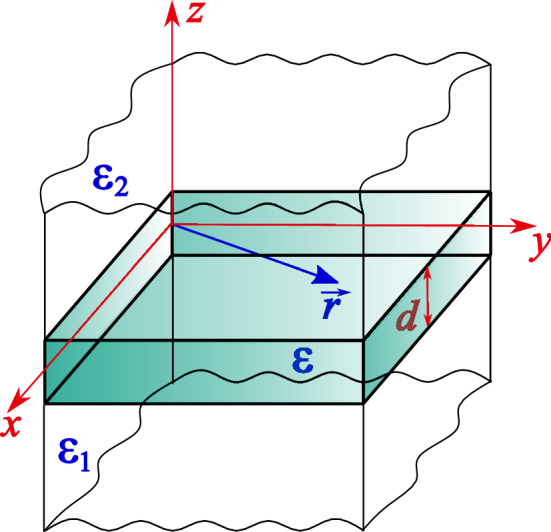
Geometry of the problem under consideration. Thin semiconducting film of thickness *d* and dielectric permittivity $\varepsilon$ is placed between substances with dielectric constants $\varepsilon _1$ and $\varepsilon _2$. 2D radius-vector ${\mathbf{r}}$ in the *xy* plane is also shown.

As we pointed out above, the dielectric screening of Coulomb interaction plays the most fundamental role in semiconductors, determining, for instance, the exciton binding energy. On the other hand, it has been shown^27^ that in 2D case the screening, determined by Rytova–Keldysh interaction^28,29^, is spatially nonlocal, which in the disordered case gives many nontrivial effects. In the present paper we study the synergy between disorder and dielectric screening in 2D semiconductor structures. For that we consider a 2D hydrogenic model with screened Coulomb potential, determined by Rytova–Keldysh interaction^28,29^. Also, in our model, all kinds of disorder are mimicked by the introduction of the fractional Laplacian in the corresponding Schrödinger equation. For instance, if a substance has a small number of almost isolated (weakly interacting and sparsely located) defects or impurities, each of them serves as a trap for excitons. The same is true for excitons localization at the different inhomogeneities like dislocations, grain boundaries, interfaces, etc. In this case, the potential of inhomogeneity (or single defect as for small concentration they can be considered non-interacting) plays a role of additional localization factor for exciton. For such kind of problems, ordinary (i.e. nonfractional) Schrödinger equation gives a pretty good description of existing experimental data^30^. On the contrary, when we are dealing with substantial substance amorphization (strong disorder), the exciton spectrum cannot be described adequately by the ordinary Schrödinger equation. Needless to say, that the synergy between dielectric screening (which is especially important in 2D dielectrics) and strong disorder may lead to unexpected outcomes.

Note that electrons in highly disordered substances are located not in an ordered crystalline potential, but in the random field of impurities. This implies that in this case, the potential is no more periodic so that Bloch’s theorem is no longer valid. Therefore, electronic states are not expected to be periodic (with ideal ordered lattice potential periodicity) Bloch functions. This means, in turn, that in amorphous substance, the band theory of solids, based on the above translational symmetry, is inapplicable, see, e.g.^31^. In other words, strong disorder destroys any symmetry of the initial (before, say, doping) crystalline structure. Thence, highly disordered (say, amorphous) semiconductors of any original symmetry behave similar to each other, resembling, say, isotropic ceramics. That is why for our purposes it is sufficient to consider the simplest possible two-band effective mass model of an exciton.

Here we consider theoretically the joint action of disorder and Coulomb interaction screening in the 2D dielectrics. The special emphasis is made on the above considered semiconductor interfaces and heterostructures.

## Disorder and heavy-tailed Lévy distributions

The most important question of the present consideration is how fractional derivatives, generating heavy-tailed (i.e. decaying slower than Gaussian, typically in a power-law fashion $x^{-1-\mu }$, where *x* is any physical quantity like energy or potential barrier height) Lévy distributions, are related to the disorder. The common wisdom is that disorder is a lack of regularity. In this case, the above physical quantities are not under precise control so that the properties of such disordered systems are described in terms of distribution functions. The goal now is to predict global properties shared by almost all such systems, i.e. to acquire knowledge of universal features independent of the precise realization of the disorder. If the disorder is weak (i.e. a crystal has a small number of noninteracting defects and/or impurities), its properties are well described by the Gaussian distribution function. As this distribution falls off rapidly, its width is usually modest so that uncommon, “highly disordered” configurations have (very) small statistical weights and do not contribute to the observable properties of such (ordered or weakly disordered) systems. In a highly disordered material, atoms are not arranged in crystalline periodic patterns but appear in random positions. This means that the actual statistical weight of the above “highly disordered” configurations grows, sometimes enormously. This large statistical weight is indeed described by the above non-Gaussian, heavy-tailed distributions. As we discussed above, in an amorphous substance, the electronic states are no more Bloch functions due to severe violation of the translational symmetry. The simplest model that describes the electronic states in the highly disordered matter was introduced by Anderson^32^ and leads to the famous Anderson localization phenomenon.

Namely, the main theorem of Anderson^32^ states that if the breadth of energy distribution *W* is around mean value *V* of the interaction potential between disorder constituents (i.e. the particles like electrons, excitons, spins) located at the sites of some lattice, the transport in such system is absent. Note that to this end, the specific form of the above energy distribution is not given, the only condition is that it should be sufficiently broad. In other words, in a system with the aforementioned strong disorder, having a wide enough distribution of its physical characteristics, all states are strongly localized. In our context, the above “disordered lattice” means considerable substance amorphization or other types of strong disorder, when defects or impurities, having large concentration, start to strongly interact with each other. Such a strong disorder implies that the above distribution function of the energy or any other physical characteristic is strongly non-Gaussian. At this point, it is reasonable to assume that this distribution is Lévy one.

The next observation is that the Eq. (1) of seminal Anderson’s paper^32^ is very similar (with respect to substitution $t \rightarrow it$, *i* is an imaginary unit) to Langevin equation, governing the stochastic dynamics of the (in our case two-dimensional) coordinate ${\mathbf{r}}(t)$ of a particle

1 $$\begin{aligned} \frac{d{\mathbf{r}}(t)}{dt}=-\frac{\nabla V({\mathbf{r}})}{m\gamma }+{\mathbf{z}}(t). \end{aligned}$$

Here *m* is a particle mass and $\gamma$ is a friction coefficient. Also, ${\mathbf{z}}(t)$ is a noise function, responsible for stochastic behavior. Once more, we assume that ${\mathbf{z}}(t)$ obeys Lévy statistics with probability distribution, most conveniently defined by its Fourier image or characteristic function $f(k)=\exp (-\sigma ^\mu k^\mu /\mu )$, where $k\equiv |{\mathbf{k}}|$ and $0<\mu <2$ is Lévy index, see^22,23,33^ and references therein. The case $\mu =2$ corresponds to Gaussian distribution with variance $\sigma$. This characteristic function is valid for any space dimensionality. In 2D it generates following explicit expression

2 $$\begin{aligned} f(r)=\frac{1}{2\pi }\int _0^\infty J_0(kr)\exp \left[ -\frac{\sigma ^\mu k^\mu }{\mu }\right] kdk, \end{aligned}$$

where $J_0(x)$ is Bessel function^34^. Note that the distribution (2) at $\mu <2$ have an infinite variance as the corresponding integral becomes divergent. For such heavy-tail distribution to have higher moments, we need some external potential (like in Fokker–Planck or Schrödinger equation), which “tames” the corresponding Lévy flight (common name for the processes, described by distribution (2)), see^35^ and references therein. Both Eq. (1) of the paper^32^ and Langevin equation (1) are stochastic differential equations. The probability density $p({\mathbf{r}},t)$ for the Eq. (1) is given by the (fractional for $\mu <2$) Fokker–Planck equation

3 $$\begin{aligned} \frac{\partial p({\mathbf{r}},t)}{\partial t}=\nabla \left[ \frac{p({\mathbf{r}},t)\nabla V({\mathbf{r}})}{m \gamma }\right] -D|\Delta |^{\mu /2}p({\mathbf{r}},t). \end{aligned}$$

Here $|\Delta |^{\mu /2}$ is the fractional Laplacian, which in two dimensions reads

4 $$\begin{aligned} -|\Delta |^{\mu /2}f({\mathbf{x}})= & {} A_\mu \int \frac{f({\mathbf{u}})-f({\mathbf{x}})}{|{\mathbf{u}}-{\mathbf{x}}|^{\mu +2}}d^2u, \end{aligned}$$

5 $$\begin{aligned} A_\mu= & {} \frac{2^\mu \Gamma \left( \frac{\mu +2}{2}\right) }{\pi |\Gamma (-\mu /2)|}. \end{aligned}$$

Here $\Gamma (x)$ is $\Gamma$—function^34^, ${\mathbf{r}}$ is a two-dimensional vector. For $\mu =2$ the operator (4) gives ordinary 2D Laplacian^36^. The details of the derivation of fractional Fokker–Planck equation (2) from Langevin equation with Lévy noise (1) can be found, for instance, in Ref.^37^. Such derivation reduces to the “extraction” of probability density from the noise characteristics. This “extraction” procedure is also present both in the derivation of ordinary Schrödinger equation from Feynman path integral^38^ and in the derivation of fractional one by Laskin using the same construction but with Lévy measure^26^. Moreover, the free versions (at potential $V=0$) of both equations can be reduced to each other by obvious transformations $t \rightarrow it$ and $D=\hbar ^2/(2m)$. This means that we can safely assume that the substitution of the underlying Gaussian distribution by slowly decaying, heavy-tailed one in the Schrödinger equation is equivalent to phenomenological account for (strong) disorder. In this case, the Lévy index $\mu$ serves as an indicator of the degree of disorder. Note that such suppositions have been made in Ref.^39^ in the context of spectral narrowing of nuclear magnetic resonance lineshape. The seminal paper of Sher and Montroll^40^ is dealing actually with the same situation. Here we note one more pioneering work^41^, considering the mesoscopic phenomenon of conductance of 1D quantum wire with the strong disorder. Namely, the randomness in the scatterers ensemble had Lévy distribution. It had been shown in Ref.^41^ that the conductance statistics in this case is governed primarily by the Lévy index (the scaling exponent in the notations of^41^), which also signifies the degree of disorder.

**Figure 2:**
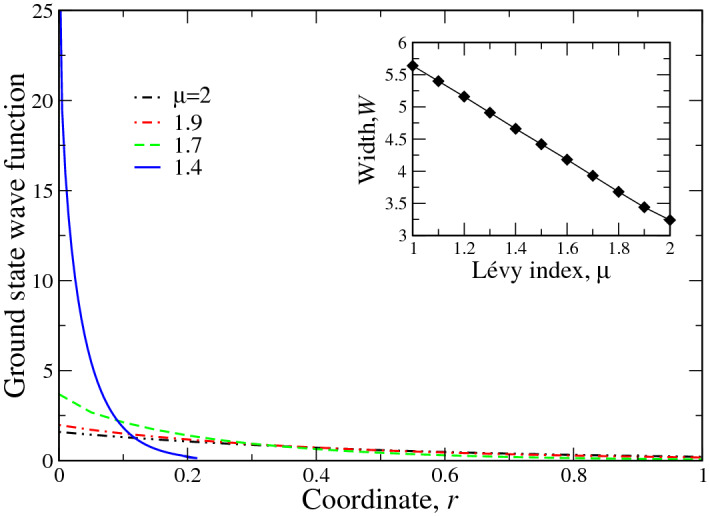
Shown in the main panel is the radial part of ground state wave function for dimensionless screening radius $\xi =0.1$ (see Eq. (27)) and different Lévy indices $\mu$, shown in the legend. It is seen that as Lévy index decreases from $\mu =2$ (corresponding to the case of ordinary Laplacian in the Schrödinger equation) to $\mu =1.4$, the ground state wave function becomes progressively more localized. It can be shown (see below) that at $\mu \rightarrow 1$ the wave function tends to Dirac $\delta$ function. The inset reports the breadth of initial Lévy distribution (2) as the function of Lévy index $\mu$. The breadth has been taken at the point, when the corresponding Gaussian (Lévy distribution at $\mu =2$) normalization equals to 0.999. This approximately corresponds to 3$\sigma$ in Eq. (2), where $\sigma =1$.

The pictorial demonstration of this fact is shown in Fig. 2, where the radial part $\psi _{00}(r)$ of the ground state wave function of the 2D screened fractional hydrogen atom is reported. The main panel of Fig. 2 shows the progressive localization of $\psi _{00}(r)$ at Lévy index diminishing. At the same time, the inset shows the width *W* of initial Lévy distribution (2). The comparison of the inset and main panel of Fig. 2 demonstrates that as initial width *W* increases, the wave function becomes more localized, tending to Dirac $\delta$ function as $\mu \rightarrow 1$. This shows that for our system the conditions of Anderson theorem^32^ is fulfilled, which supports our assumption that the introduction of fractional Laplacian in the Schrödinger equation effectively describes disorder. Note, that the breadth of 1D Lévy distribution, realizing for scalar quantities like energy, behaves qualitatively similar to the inset in Fig. 2. Moreover, our preliminary studies of continuous spectrum in the problem under consideration show that the corresponding wave functions start to localize at $\mu \approx 1.9$, which is also related to the disorder. This means that as $\mu$ deviates from 2, the lower bound of the continuous spectrum starts to shift so that at $\mu \rightarrow 1$ only the small portion of continuous spectrum (if any at all) may remain. The answer to this question is so far unclear as we are dealing with the 2D system. It is well known (see, e.g.^42^) that in the 1D system at strong disorder all states are localized. This problem, however, is out of the frame of present consideration and will be published elsewhere.

## Theoretical approach

To model the problem of an exciton in 2D disordered dielectric, here we consider 2D fractional (mimicking disorder) hydrogenic problem, where the exciton “resides” in the *xy* plane. Geometry of our problem is shown in Fig. 1. The question about the form of interaction potential *U*(*r*) in the structure, shown in Fig. 1, is usually thought of as a solution of Poisson equation (see, e.g.^43^) $\Delta U({\mathbf{r})}=-4\pi \rho ({\mathbf{r}})$, where $\rho ({\mathbf{r}})$ is a total charge density at the point ${\mathbf{r}}$, which is responsible for screening of the Coulomb interaction. The explicit form of the screened interaction potential *U*(*r*) had been found by Rytova^28^ and subsequently by Keldysh^29^. It reads

6 $$\begin{aligned} U(r)=-\frac{\pi \beta }{2r_0}\left[ H_0\left( \frac{r}{r_0}\right) -Y_0\left( \frac{r}{r_0}\right) \right] , \end{aligned}$$

where $r=|{\mathbf{r}}|$, $\beta =e^2/\varepsilon$ (*e* is electronic charge, $\varepsilon$ is the dielectric permittivity of middle layer in Fig. 1),

7 $$\begin{aligned} r_0=\frac{\varepsilon d}{\varepsilon _1 +\varepsilon _2} \end{aligned}$$

is the screening radius, see Fig. 1. Here $H_0(z)$ and $Y_0(z)$ are Struve and Neumann functions respectively^34^. At zero screening $r_0 \rightarrow 0$, the argument $z=r/r_0$ of the functions $H_0(z)$ and $Y_0(z)$ tends to infinity so that^34^

8 $$\begin{aligned} \left. H_0(z)-Y_0(z)\right| _{z \rightarrow \infty }\approx \frac{2}{\pi z} \end{aligned}$$

and

9 $$\begin{aligned} \left. U(r)\right| _{r_0 \rightarrow 0}= -\frac{\beta }{r}, \end{aligned}$$

which corresponds to the ordinary 3D Coulomb interaction. As demonstrated in^43^, in 2D layers of finite thickness, the solution of the Poisson equation has the above 1/*r* form (rather than well-known logarithmic law, inherent in 2D systems^44^) due to the presence of a hole (which, say, is our exciton “component”) at the origin. The case of fractional 2D exciton in the layers of finite thickness with unscreened interaction (9) has been considered in Ref.^45^.

At $r_0 \rightarrow \infty$, the argument of the functions in (6) $z=r/r_0 \rightarrow 0$. The asymptotics reads^34^

10 $$\begin{aligned} \left. H_0(z)-Y_0(z)\right| _{z \rightarrow 0}\approx -\frac{2}{\pi }\left( \gamma + \ln \frac{z}{2}\right) , \end{aligned}$$

where $\gamma \approx 0.5772$ is the Euler’s constant^34^. It is easy to see that this asymptotics corresponds to ordinary 2D Coulomb interaction in the form $\ln r$, signifying the Green’s function of 2D Poisson equation^44^. In other words, the Rytova–Keldysh interaction (6) gives the transition from ordinary 2D Coulomb interaction $\ln r$ in the case of infinite screening $r_0 \rightarrow \infty$ to the case of 3D one (“ordinary” Coulomb $\sim 1/r$) at zero screening $r_0 \rightarrow 0$.

Having potential (6), we can write down the fractional Scrödinger equation for our 2D fractional hydrogenic problem. It reads

11 $$\begin{aligned}&A_\mu \int \frac{\Psi _{nm\mu }({\mathbf{u}})-\Psi _{nm\mu }({\mathbf{r}})}{|{\mathbf{u}}-{\mathbf{r}}|^{\mu +2}}d^2u +U(r)\Psi _{nm\mu }({\mathbf{r}})\nonumber \\&\quad =E_{nm\mu }\Psi ({\mathbf{r}}), \end{aligned}$$

where *U*(*r*) is defined by Eq. (6) and $A_\mu$ by (5). The Eq. (11) is a (singular as the integral in it exists in the sense of Cauchy principal value only) two-dimensional integral equation with fractional Laplacian having Lévy index $\mu$. Latter index, in a sense, defines how far the fractional Laplacian deviates from ordinary one so that the eigenvalue problem (11) deflects from conventional 2D hydrogenic one^46^. Note that if we define the fractional Laplacian (4) in 3D (1D) space, the Eq. (11) with properly tailored potential (that is to say, 3D or 1D Coulomb interaction) will give 3D (1D) fractional hydrogenic problem.

Indices *n* and *m* denote, respectively, the principal and orbital quantum numbers, which are different for any specific $\mu$ value. Below we shall see that for $\mu <2$, the so-called Coulomb degeneracy is lifted and that is the reason why the eigenenergy *E* has now two subscripts. To be more specific, it can be shown that in the fractional case, the “accidental” Coulomb degeneracy (stemming from Runge–Lenz vector conservation for $\mu =2$^47,48^) is lifted so that the eigenenergy starts to depend on the orbital quantum number. Note, that this feature does not preclude the rotational (around *z* or $k_z$ axis in our case) invariance of the Schrödinger equation so that its solutions can still be separated on the radial and angular parts.

Here we use modified (for the fractional case $\mu <2$) Rydberg units^26^, i.e. we measure the energy *E* and coordinates ${{{\mathbf {r}}}}$ in the units

12 $$\begin{aligned} E_{0\mu }=\left( \frac{\beta }{2\hbar }\right) ^{\frac{\mu }{\mu -1}}D_\mu ^{-\frac{1}{\mu -1}},\ r_{0\mu }=\left( \frac{2\hbar ^\mu D_\mu }{\beta }\right) ^{\frac{1}{\mu -1}} \end{aligned}$$

respectively. Parameter $\beta =e^2/\varepsilon$, while $D_\mu$ is a mass term^26^. At $\mu =2$ units (12) convert into standard Rydberg units. Note that at $\mu =1$ both $E_{01}$ and $r_{01}$ in Eq. (12) are divergent. This is one more manifestation of the fact, that discrete spectrum of 2D quantum fractional hydrogenic problem exists at $\mu >1$ only^45^.

The Eq. (11) is indeed an integral equation, sometimes called pseudo-differential one^33^. Moreover, the integral (4) exists only in the sense of Cauchy principal value^33,36^, which is a source of additional difficulties in such equations solutions. To bypass this, here we transit to ${\mathbf{k}}=(k_x,k_y)$ space as the operator (4) becomes $-k^\mu$, where $k\equiv \sqrt{k_x^2+k_y^2}$. Although the second term in (11) converts to the integral in momentum space, it proves to be much easier to handle then initial one (4). This becomes clear if we consider the Fourier image of the potential *U*(*r*), which is much simpler, then initial expression (6):

13 $$\begin{aligned} U(k)=-\frac{2\pi \beta }{k(1+kr_0)}. \end{aligned}$$

The particularly simple expression (13) permits to do the angular integration exactly, i.e. without expansion over spherical harmonics, see^49^ and references therein. Latter fact permits us to solve the corresponding fractional Schrödinger equation by the method similar to the case without screening^45^.

In dimensionless variables (12), the Fourier image of the Scrödinger equation (11) assumes the form

14 $$\begin{aligned} (k^\mu -E)\Psi ({\mathbf{k}})+\frac{1}{(2\pi )^2}\int V(|{\mathbf{k}}-{\mathbf{k}}'|)\Psi ({\mathbf{k}}')d^2k^{\prime}=0, \end{aligned}$$

where $V(q)=-4\pi /[q(1+qr_0)]$ is a dimensionless version of the Fourier image (13). Substitution of this image into the Eq. (14) generates following form of the Eq. (11) in momentum space

15 $$\begin{aligned} (k^\mu +k_0^\mu )\Psi ({\mathbf{k}})-\frac{1}{\pi }\int \frac{\Psi ({\mathbf{k}}')d^2k^{\prime}}{q(1+qr_0)}=0, \end{aligned}$$

16 $$\begin{aligned} q=|{\mathbf{k}}-{\mathbf{k}}'|=\sqrt{k^2+k^{\prime 2}-2kk^{\prime}\cos (\varphi -\varphi ')}, \end{aligned}$$

where $\varphi$ and $\varphi '$ are asimuth angles of the vectors ${\mathbf{k}}$ and ${\mathbf{k}}'$ respectively. Here, generalizing the case of ordinary ($\mu =2$) 2D hydrogen atoms^49^, we denote

17 $$\begin{aligned} E=-k_0^\mu . \end{aligned}$$

It can be seen that at $\mu =2$ and $r_0=0$, Eq. (15) gives the well-known momentum space representation of 2D hydrogenic problem with $\mu =2$ and unscreened “sheet” Coulomb interaction (9)^49^.

For any central (i.e. spherically symmetric) force potential (which is also the case for potential (13)), the angular and radial variables (in ${\mathbf{k}}$ space in our case) can be separated so that we can look for a solution of the Eq. (15) in the form

18 $$\begin{aligned} \Psi _{nm\mu }({\mathbf{k}})=\psi _{nm\mu }(k)e^{im\varphi }. \end{aligned}$$

As usual in hydrogenic problems, radial functions $\psi _{nm\mu }(k)$ are real. The normalization condition for these functions read

19 $$\begin{aligned} 2\pi \int _0^\infty \psi _{nm\mu }^2(k)kdk=1. \end{aligned}$$

Substitution of *ansats* (18) into Eq. (15) yields

20 $$(k^{\mu} +k_{0}^{\mu })\psi (k)+\frac{1}{\pi }\int _{0}^{\infty} I_m(k,k^{\prime}) \psi (k^{\prime}) k^{\prime}dk^{\prime}=0,$$

21 $$I_m(k,k^{\prime})=\int _{\varphi }^{\varphi -2\pi }\frac{{e^{-imt}}dt}{q_t(1+q_tr_0)}, $$

22 $$ q_t= \sqrt{k^2+k^{\prime 2}-2kk^{\prime}{\cos} t}. $$

Here for clarity, we suppress the subscripts $n, \mu$.

Note that in “ordinary” (non-fractional) 3D quantum hydrogenic problem, the corresponding Scrödinger equation is usually solved by stereographic projection method, which is due to Fock^48^ (see also Ref.^49^ for 2D case and Ref.^50^ for multidimensional case). In this method, the problem can be solved exactly using spherical harmonics expansion^48–50^. It can be shown that in 2D screened case (13), such stereographic projection is impossible. The same is true for fractional case $\mu <2$. It turns out, however, that the simple form of Fourier image of Rytova–Keldysh potential (13) admits the possibility of exact analytical calculation of the integrals $I_m(k,k^{\prime})$ (21) for each specific *m*. This permits to easily solve the problem numerically as it now reduces to the effective one-dimensional integral equation^51^. The integral for $m=0$ yields

23 $$\begin{aligned} I_0(k,k^{\prime})= & {} \int _{\varphi }^{\varphi -2\pi }\frac{dt}{q_t(1+q_tr_0)}=-\frac{4}{k+k^{\prime}}\ \nonumber \\&\times \frac{1}{1-(k+k^{\prime})^2r_0^2}\ \Pi \left( \frac{4kk^{\prime}r_0^2}{(k+k^{\prime})r_0^2-1},\frac{4kk^{\prime}}{(k+k^{\prime})^2}\right) , \end{aligned}$$

where $\Pi (m,n)$ is a complete elliptic integral of the third kind^34^. The asymptotics of the integral $I_0(k,k^{\prime})$ (23) for unscreened case $r_0=0$ has the form^45^

24 $$\begin{aligned} I_{0\ Coul }=-\frac{4}{k+k^{\prime}}K\left( \frac{4kk^{\prime}}{(k+k^{\prime})^2}\right) , \end{aligned}$$

where *K*(*m*) is a complete elliptic integral of the first kind^34^. Note that at $k=k^{\prime}$ the argument of the complete elliptic integral in (24) equals to 1, which implies that *K* is divergent. This reflects the spherical harmonic series divergence at $k=k^{\prime}$ for unscreened case. In the screened case (23) the situation is more complicated as function $\Pi (m,n)$ is divergent at both $m=1$ and $n=1$. However, as it can be shown, these divergences are well compensated by other parts of corresponding integrands. We note also, that function $\Pi (m,n)$ has different representations at different values of its parameters, see Ref.^34^ for details. This fact should be taken into account in numerical calculations.

The expressions for the integrals $I_m(k,k^{\prime})$ at arbitrary *m* can also be obtained in closed form, although the expressions for higher *m*’s become more and more cumbersome. After lengthy calculations, we arrive at the following form for $I_1(k,k^{\prime})$

25 $$\begin{aligned} I_1(k,k^{\prime})= & {} -\frac{2}{kk^{\prime}(k+k^{\prime})r_0^2}\Bigg \{\frac{(k^2+k^{\prime 2})r_0^2-1}{1-(k+k^{\prime})^2r_0^2}\nonumber \\&\times \Pi \left( \frac{4kk^{\prime}r_0^2}{(k+k^{\prime})^2r_0^2-1},\frac{4kk^{\prime}}{(k+k^{\prime})^2}\right) \nonumber \\&+K\left( \frac{4kk^{\prime}}{(k+k^{\prime})^2}\right) \Bigg \}. \end{aligned}$$

The set of Eq. (20) define (for each specific *m*) the spectrum of fractional 2D screened hydrogen atom for that particular *m* and all $n\ge m$. To be specific, the equation for $m=0$ (see (20) and (23)) defines the wave functions $\psi _{00\mu }$ (ground state), $\psi _{10\mu }$, $\psi _{20\mu }$ etc. The equation for $m = 1$ determines $\psi _{11\mu }$, $\psi _{21\mu }$, etc. The same is true for higher *m*.

Equation (20) is the main theoretical result of the present consideration. As we shall see below, it is especially useful in our case of screened Coulomb interaction as it reduces the problem solution to the 1D integral equation, which numerical solutions behave well, see below.

## Solution of the spectral problem

Let us pass to numerical solution of the set (20) for each specific *m*. For that we introduce $k/k_0=x$, $k^{\prime}/k_0=y$, which yields

26 $$\begin{aligned} \kappa _0\psi _{nm\mu }(x)=-\frac{1}{\pi }\frac{1}{x^\mu +1} \int _0^\infty I_m(x,y)\psi _{nm\mu }(y)ydy, \end{aligned}$$

where $\kappa _0=k_0^{\mu -1}$ so that the eigenenergy $E=-\kappa _0^{\frac{\mu }{\mu -1}}$. The functions $I_m(x,y)$ are defined by the expression (21). The Eq. (26) has been solved numerically by discretization and subsequent solution of the spectral problem for the obtained matrix, see Ref.^51^ for details. The explicit form of the equation for $m=0$ reads

27 $$\begin{aligned} \kappa _0\psi _{n0}(x)= & {} \frac{4}{\pi }\frac{1}{x^\mu +1}\int _0^\infty \frac{y}{x+y}\ \nonumber \\&\times \frac{\Pi (n,m)\ \psi _{n0}(y)}{1-\xi ^2 (x+y)^2}dy,\ \xi =k_0r_0,\nonumber \\ n= & {} \frac{4\xi ^2 xy}{\xi ^2 (x+y)^2-1},\ m=\frac{4xy}{(x+y)^2}. \end{aligned}$$

The same for $m=1$ has the form

28 $$\begin{aligned} \kappa _0\psi _{n1}(x)= & {} \frac{2}{\pi }\frac{1}{x(x^\mu +1)\xi ^2}\int _0^\infty \frac{\psi _{n1}(y)}{x+y}\nonumber \\&\times \bigg \{\frac{\xi ^2 (x^2+y^2)-1}{1-\xi ^2 (x+y)^2}\ \Pi (n,m)+K(m)\bigg \}dy, \end{aligned}$$

where the parameters $\xi$, *n* and *m* are defined by (27).

**Figure 3:**
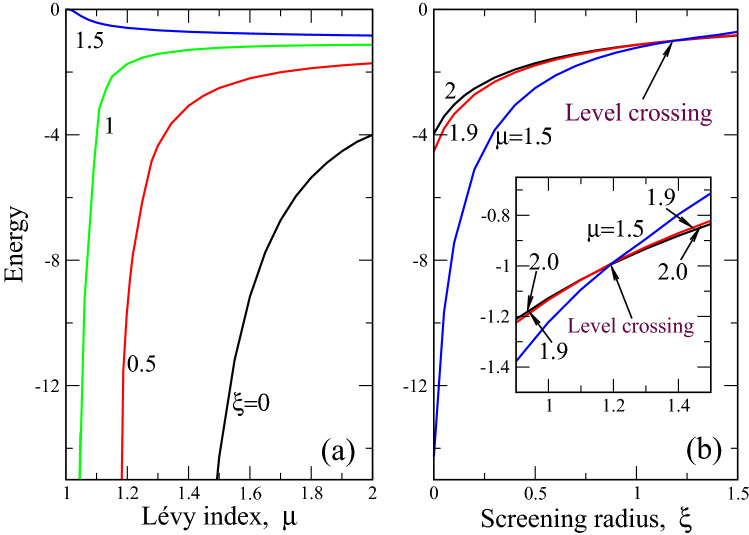
Ground state energy (in the units $E_{0\mu }$ (12)) as a function of Lévy index $\mu$ (**a**) and dimensionless screening radius $\xi$ (b). Figures near curves define the screening radius (panel (**a**)) and Lévy index (panel (**b**)). Inset to panel (**b**) details the behavior of ground state energy at $\xi >0.9$, where energy levels crossing occurs approximately at $\xi \approx 1.15$. Levels crossing point is the same for all $1<\mu <2$ and occurs for finite screening only.

It is instructive to consider the known case of “ordinary” (i.e. with normal Laplacian in the Schrödinger equation) 2D hydrogen atom with unscreened Coulomb interaction. Such spectral problem is well studied (see^46^ for coordinate space and^49^ for momentum space) and its energy spectrum in our Rydberg units reads

29 $$\begin{aligned} E=-\frac{1}{(n+1/2)^2},\ n=0,1,2,3,... \end{aligned}$$

**Figure 4:**
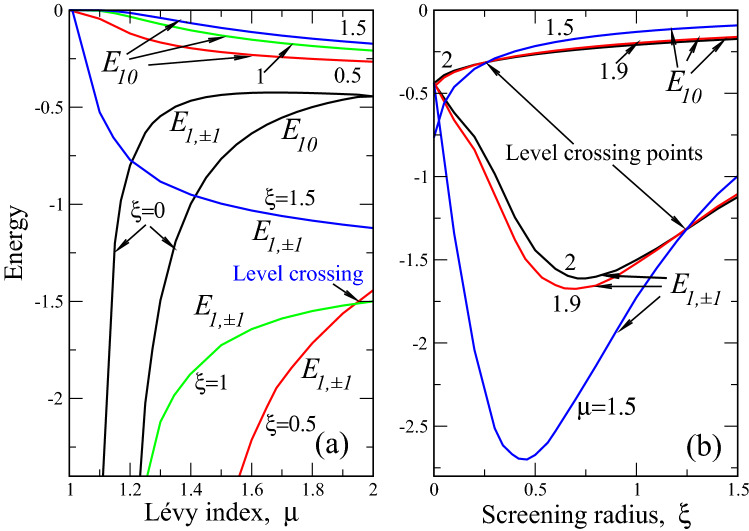
Same as in Fig. 3 but for the first excited state $E_{10}$ and $E_{1,\pm 1}$. Lifting of orbital degeneracy, related to Runge–Lenz vector conservation at $\mu =2$, is seen for entire $\mu$ and $\xi$ domains (except one point $\xi =0$, $\mu =2$, at which $E_{00}=E_{1,\pm 1}=0.4444$ (29)), where $E_{00}\ne E_{1,\pm 1}$. The curves $E_{00}$ and $E_{1,\pm 1}$, corresponding to the same $\xi$ (panel (a)) or $\mu$ (panel (b)), are coded by the same color (black for $\xi =0$, red for $\xi =0.5$, green for $\xi =1$ and blue for $\xi =1.5$ in panel (a) as well as black for $\mu =2$, red for $\mu =1.9$ and blue for $\mu =1.5$ in panel (b)) and also by figures near curves. As dependencies of $E_{00}$ and $E_{1,\pm 1}$ on screening radius $\xi$ are nonmonotonous (see panel (b)), we show explicitly which curves correspond to $E_{00}$ and which to $E_{1,\pm 1}$ in both panels. The positions of level crossing points are also shown.

Figure 3 shows the ground state energy (proportional to exciton binding energy) as a function of Lévy index $\mu$ (a) and dimensionless screening radius $\xi$ (b). Similar to the case without screening^45^, the discrete spectrum in our problem exists at $1<\mu <2$. Also, at $\mu =2$ and $\xi =0$ (case of “ordinary” 2D quantum mechanical hydrogen atom) the exciton ground state energy equals to -4, which follows from Eq. (29). It can be shown that at $\xi =0$ and $\mu =2$ our numerical calculation gives correct values for the entire spectrum (29) within 1% accuracy. This fact can be thought of as a consistency check for our numerical solution.

It is seen from Fig. 3a that for relatively small screenings $0<\xi <1.5$ the ground state energy goes to minus infinity at $\mu =1$. At the same time at $\xi =1.5$, this energy goes to zero which means that strong screening “kills” the bound state between electron and hole in an exciton. Our analysis shows that this is the consequence of the competition between disorder (defined by fractional Laplacian) and screening effects. Namely, while the former deepens the “potential well”, where the bound state exists (say, it promotes the bound state), the latter (at large screenings) makes this well become shallow so that the bound state becomes progressively “less bound” as $\xi$ increases. It can be shown that at $\xi \le 1.42$ the ground state energy goes to minus infinity at $\mu =1$ (weak screening, disorder prevails, making the exciton particularly stable), while at $\xi > 1.42$ (strong screening) the situation is opposite, which is shown by the curve for $\xi =1.5$. At $\xi >1.5$ the ground state energy in the entire domain $1<\mu <2$ tends to zero.

The second important effect is energy levels crossing^52^, which is seen in Fig. 3b. The crossing occurs around $\xi =1.18$ for all admissible $1<\mu <2$. That is to say, in the point $\xi =1.18$ we have the infinitely degenerate (for all continuously varying $1<\mu <2$ ) ground state energy of an exciton in a 2D system. As the ordered case corresponds to the only $\mu =2$, we can assert that the energy level crossing in our system occurs as a result of the synergy between disorder (mimicked by fractional Laplacian with Lévy index $\mu$) and screening. Note that there is no energy levels crossing without screening in the disordered 2D excitons^45^.

Figure 4 portrays the energies of the first excited state, corresponding to $n=1$ and $m=0,1$. The main effect here is lifting of orbital degeneracy due to nonconservation of the Runge–Lenz vector^47^. To be specific, if for $\mu =2$ and $\xi =0$, the energy is independent of orbital quantum number *m* (see Eq. (29)), this is not true for $\mu <2$ (disorder) and $\xi \ne 0$ (finite screening). In other words, in our problem, the Runge–Lenz vector nonconservation comes from both disorder and finite screening effects. This fact should be taken into account while designing optoelectronic devices based on disordered heterostructures, interfaces and other 2D semiconductor structures. Note, that nonconservation of Runge–Lenz vector does not mean the nonconservation of *z* -projection of angular momentum, i.e. central symmetry of the problem. Latter conservation law is reflected in the independence of energy of the *sign* of orbital index *m*, i.e $E_{n,m}=E_{n,-m}$; it is lifted, when time inversion symmetry is broken by, e.g., external magnetic field^47^.

The energy level crossing is also present for the states with $n=1$. They occur both for different $\xi$ at $\mu \approx 1.95$ (Fig. 4a) and different $\mu$ at $\xi \approx 0.3$ for energy level $E_{10}$ as well as $\xi \approx 1.25$ for energy level $E_{1,\pm 1}$, Fig. 4b. The electron-hole bound state destruction by strong screening is also present in the first excited state, although the picture is more diverse (then in the ground state) as now orbital index *m* comes into play. Namely, if the energies $E_{10}$ are less susceptible to screening (they go to zero at $\mu =1$ for quite strong screening $\xi =1.5$), the levels $E_{1,\pm 1}$ go to zero already at $\xi =0.5$, showing higher sensitivity to screening effects. This feature becomes especially notable at strong disorder around $\mu =1$, where (for instance for small screenings $\xi =0.5$) the energy $E_{10}$ goes to zero, while $E_{1,\pm 1}$—to minus infinity. This means that at strong disorder and finite screening, our 2D excitons are more stable for higher values of orbital quantum number *m*. Our numerical analysis shows that this effect realizes for higher excited states also. Of course, for higher *n* this effect is even more diversified as now we have all $m<n$ at our disposal. The latter feature is also important for the proper functioning of different optoelectronic devices, based on disordered 2D structures. It can also be shown, that energy level crossing (having again much more crossing points than for $n=1$) occurs also for higher $n>1$.

One more interesting feature is seen in Fig. 4b. Namely, while $E_{10}$ grows monotonously at screening radius $\xi$ increase, the energy $E_{1,\pm 1}(\xi )$ is notably nonmonotonous having minimum around $\xi =0.5$, which depends on Lévy index $\mu$. The higher is $\mu$ deviation from 2, defining the degree of disorder, the deeper is minimum. In other words, we see that the branch $E_{1,\pm 1}$ has lower energy than that of exciton in the ordered 2D model without screening. It can be shown that this feature occurs also for $E_{2,\pm 2}$, $E_{3,\pm 3}$ and possibly higher excited states. This shows that the synergy between disorder and (nonlocal^27^) screening in two dimensions minimizes the energy (as compared to the ordered unscreened case) for some values of orbital quantum number *m*. This effect is also very important for the devices, using transitions (like dipole ones between *m* and $m \pm 1$) between levels with different *m*’s.

The remark about the validity of our fractional hydrogenic model is in place here. We recollect Laskin’s construction of fractional quantum mechanics^26^, which uses Feynman path integration but with Lévy measure. Since the tails of Lévy distributions are much “heavier” than Gaussian, the corresponding distribution function (which in resulting Schrödinger equation yields a wave function) spans over many lattice constants. In the preceding discussion, we have shown that one of the sources of Lévy (rather than Gaussian) distributions is strong disorder, i.e. the situation when almost all lattice sites are occupied by impurities and the lattice by itself is highly distorted. In this case, Laskin’s construction generates the “enveloping function” giving a good average description of such systems. In that sense our model, based on fractional quantum mechanics, presents the result of “smearing” the microscopic disorder in a 2D semiconductor, representing it as some effective medium. The price which we pay for that is the substitution of the ordinary Laplacian by its fractional counterpart in the corresponding Hamiltonian. Note, that the present approach is complimentary to microscopic one^53,54^, where the explicit averaging over disorder had been performed within the so-called fluctuating field method. There, for the case of strong disorder, the distribution function also appeared to be non-Gaussian. However, in the above microscopic treatment, additional suppositions about averaging over impurity configurations had been made, see Refs.^53,54^ for details. At the same time, present construction, being phenomenological by its nature, does not make any assumption about specific properties of (strong) disorder constituents.

The smallest distance between the above disorder constituents in a crystal lattice is lattice constant, which is around 5 Å. This implies that our model is valid at the distances, where the lattice discreteness is unimportant (which is true at strong disorder), i.e. around 50 Å, which is 10 lattice constants. At such distances, due to disorder averaging, also the Anderson localization^32^ occurs. Likewise, in our model, at $\mu \approx 1.2$, the disorder yields so much localization (exciton collapse), that our model breaks down. Note that the Rytova–Keldysh model also does not describe screening correctly at distances around lattice constant. This is because this model utilizes a continuous approach to the charges interaction. In this approach, the notion of dielectric permittivity is used, which is macroscopic (or at least mesoscopic such that lattice discreteness does not enter) in its nature. To be specific, in order to describe a substance in terms of its permittivities (dielectric, magnetic, etc), the effective averaging over underlying lattice discreteness should be performed^55^. In momentum space, this corresponds to small wave vectors as compared to the size of the Brillouin zone. In real space, this length-scale is different for different substances and by order of magnitude corresponds to screening radius^43^. For instance, for layered transition metals dichalcogenides like $\hbox {WS}_2$ such radius is around 40Å^56^, i.e. once more about 10 lattice constants.

Our analysis shows that the qualitative shape of the exciton wave functions is different for strong (when corresponding energy goes to zero at $\mu =1$ signifying the exciton ionization, i.e. bound state destruction, see Figs. 3, 4) and weak screenings, where, on the contrary, exciton collapses as its energy tends to minus infinity. In both screening regimes, the wave functions are sensitive to the Lévy index $\mu$. Namely, for weak screening, as $\mu$ diminishes from 2 to 1, the wave functions become progressively less localized in ${\mathbf{k}}$ space. Also, their amplitudes diminish as $\mu \rightarrow 1$ so that the wave functions become almost delocalized with zero amplitude. This situation corresponds to “extra strong” localization in coordinate space, where $\psi (r)$ degenerates into (almost) Dirac $\delta$—function, reflecting the exciton collapse, i.e. “falling” of the electron on the hole in it. The situation is opposite for strong screening, where in momentum space the wave function acquires Dirac $\delta$ function shape as $\mu \rightarrow 1$, which in coordinate space correspond to the almost delocalized wave function, corresponding to exciton ionization. Hence, the interplay between screening effects and disorder (mimicked in our approach by fractional Laplacian with Lévy index $\mu$) may lead either to exciton ionization or its collapse for the strong disorder. That is to say, the synergy between screening and strong disorder (like substance amorphization, which is common for semiconductor interfaces, where high mechanical stresses occur) may destroy (either ionize or collapse them, depending on the relation between screening radius $\xi$, Lévy index $\mu$ and orbital quantum number *m*, see above) the excitons, which may preclude the functionality of the devices like solar cells and/or light emitting diodes.

**Figure 5:**
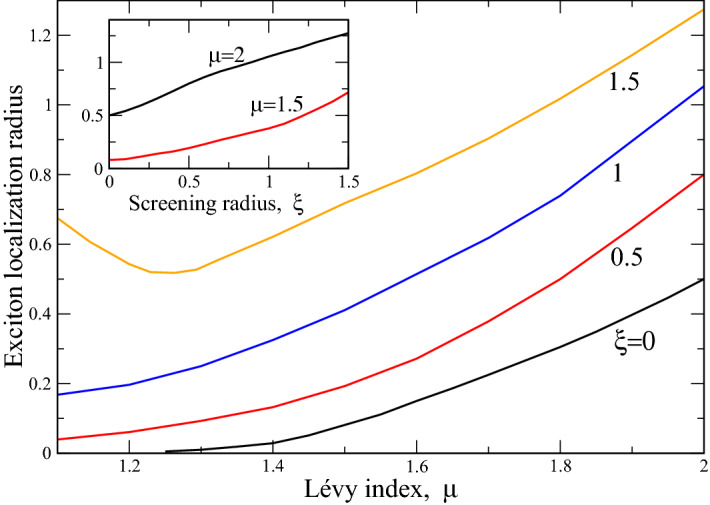
Exciton localization radius $\bar{r}$ (in the units $r_{0\mu }$ (12)) versus Lévy index $\mu$ at several values of screening radius $\xi$, shown as the figures near curves. Inset reports the dependence $\bar{r}(\xi )$ at two representative values of $\mu$, shown near curves.

## Discussion: relation to experiment

The above solution permits calculation of the observable characteristics of the excitons. One of the important characteristics is the exciton binding energy. This quantity is proportional to the exciton ground state energy and defines the work needed to remove a bound electron to infinity. The latter quantity has been already calculated and is reported in Fig. 3. One more important physical characteristic is the exciton localization radius. This parameter is a mean value of exciton radius-vector *r* in the ground state $n=m=0$

30 $$\begin{aligned} \bar{r}\equiv \bar{r}_{00\mu }=2\pi \int _0^\infty r^2\psi _{00\mu }^2(r)dr. \end{aligned}$$

To obtain the expression (30), we have integrated over the angle $\varphi$. The results of numerical calculations of $\bar{r}$ are reported in Fig. 5. It is seen that while at screening radii $\xi <1.5$ the exciton localization radius decays monotonously as $\mu$ (mimicking the degree of disorder in our problem) approaches 1, at $\xi =1.5$ it starts to grow signifying the exciton ionization at $\mu =1$. This is a reflection of the fact, that the joint action of strong screening and disorder destroys the bound state in an exciton, leading to its ionization at $\mu =1$. This effect has already been shown in Fig. 3, where ground state energy at $\xi =1.5$ and $\mu =1$ was zero. Note that at the same time, at weak screening, the disorder strengthens the bound state (this may be thought of as a kind of Anderson localization^32^), leading to exciton collapse at $\mu =1$.

To understand better the physical situation with exciton collapse, we recollect that the bound (localized) state in quantum mechanics corresponds to negative energy, $E<0$^47^. Our numerical calculations of the exciton localization radius in Fig. 5 show that the latter quantity diminishes as $\mu \rightarrow 1$, giving exactly zero (say, “infinitely localized state” of zero spatial extension) at $\mu =1$ and screening radius $\xi =0$. So, the phenomenon of exciton collapse, corresponding to an “infinitely localized state” must have infinitely large negative energy. This is actually seen in Fig. 3. Our analysis for higher excited states with $n>1$ shows the same tendency. Most probably, in real 2D structures having different kinds of disorder (say, defects and/or impurities at the interface), the exciton may be trapped and recombined before its actual collapse. The experimental and theoretical (inclusion of other sources of disorder in our model) studies of this interesting question should be carried out.

The inset of Fig. 5 portrays the dependence $\bar{r}(\xi )$ at two fixed values of Lévy index $\mu$: $\mu =2$ (ordered case) and $\mu =1.5$. In both cases, exciton localization radius grows with $\xi$ in accord with Figs. 3 and 4, where it is shown that screening breaks bound state between electron and hole in an exciton. Also, in disordered case ($\mu =1.5$) the exciton localization radius is smaller than that for $\mu =2$ for the same $\xi$. This shows that the synergy between disorder and nonlocal screening (peculiar to 2D case^27^) stabilizes the exciton. Our estimations show that for typical (not very strong, when exciton collapses) strength of disorder $\mu =1.5$, the screening radius $r_0$, corresponding to dimensionless $\xi =$1.5 is 50 Å, which is in qualitative agreement with values, known from literature sources 30 Å$<r_0<$ 80 Å^57^, see also below.

The recent discovery of high photovoltaic efficiency in organic - inorganic halide perovskites like methylammonium lead iodide ($\hbox {CH}_3 \hbox {NH}_3 \hbox {PbI}_3$)^57–59^ requires knowledge of how the disorder influences their excitonic properties, which are responsible for solar to electric power conversion in them. The exciton properties of halide perovskites are still the subject of intense research and active debate, see, e.g. refs.^60,61^. Along with aforementioned halide perovskites, other good candidates for optoelectronic applications are 2D transition metal dichalcogenides (TMD) like $\hbox {MoS}_2$, $\hbox {WS}_2$ and $\hbox {WSe}_2$^57^. In these substances, the excitons have high binding energy, which makes them extremely thermally stable. However, the influence of defects and other disorder on these excitons properties (like their relaxation and decoherence mechanisms) is still unknown to a larger extent. In the physical units, the typical exciton binding energy in 2D dichalcogenide semiconductor $\hbox {WS}_2$ is around 0.32 eV, which is much more than that (around 0.05 eV) in ordinary 3D materials^56^. This makes 2D TMD materials to be good candidates for optoelectronic and photonic devices at room temperatures. The in-plane exciton localization radius in them is of the order of 5 nm^56,57^. The numerical calculations with potential (6), performed in Ref.^56^, show that such Wannier-Mott like exciton can be well realized in atomically thin $\hbox {WS}_2$ (and other TMD^62^) monolayer with screening radius $r_0\approx 75$Å. The other sources (see^57^ and references therein) list $r_0$ to be in the range from 30 to 80 Å, obtained from *ab initio* calculations.

Discussed physical properties of an exciton in 2D materials, related to the interplay between 2D nonlocal screening and disorder can play a role in multiexciton configurations. For example, they can be relevant for interaction between distant 2D excitons in the above interfaces or semiconductor-based ultrathin films. Indeed, the exciton radii (around 5 nm, which is of the order of 10 lattice constants) presented in Fig. 5 are characterized by dipole moments $e\bar{r}$, enhancing intrinsic electric fields of the excitons and their interactions. On the other hand, these fields will be screened nonlocally so that many defects and impurities will fall in the span of exciton radius. As we have mentioned above, in the semiconductor structures with different degrees of disorder (different Levy indices $\mu$ in our formalism) such a random screened exciton–exciton interaction may lead either to their ionization (high screenings) or to the collapse (low screening) at $\mu =1$. This for sure will have a detrimental effect on the optoelectronic and/or spintronic device functionalities.

## Conclusions

The message of the present paper is that the synergy between the nonlocal screening of 2D Coulomb interaction and disorder in semiconducting (generally speaking dielectric) surfaces, interfaces, thin films, and multilayers has novel properties, which do not occur either in 2D unscreened ordered case or in 3D one. Our main supposition here is that Laskin’s construction of path integrals with Lévy measure^26^ is equivalent to “extraction” of probability density function from fractional Langevin equation and, in turn, to the assumptions made in seminal Andeson paper^32^. This (along with the fact that as the width of initial distribution grows, the exciton wave function becomes more and more localized, see Fig. 2) permits us to assert that fractional Schrödinger equation accounts for disorder phenomenologically with Lévy index $\mu$ being the measure of the degree of disorder. The fact that initial Lévy distributions do not have higher moments of order $\alpha >\mu$^22,23,33^ show that such construction describes the systems with broad (wider then Gaussian) distribution of its “Brownian paths” in a generalized Lévy sense. It is almost sure that the physical origin of such broad distribution in solids is disorder. The presence of potential *U* in the system “tames” the initial Lévy distribution, making it decay faster than that in a corresponding free problem^35,37^. In other words, here once more we have an interplay between the “breadth of disorder distribution”^32^ and system potential, which makes the probability distribution (square of modulus of the corresponding wave function) decay faster in space. This makes the problem of a fractional Schrödinger equation resemblant to Anderson localization in disordered systems.

In this context, the very interesting work in mesoscopic physics^41^ should be mentioned. This work pioneers the application of Lévy (rather than Gaussian, which are always employed in these kind of problems) distributions for the calculation of conductance through disordered quantum wires.It had been shown in^41^, that the distribution of conductances is entirely due to standard average $<\ln G>$ (*G* stands for conductance) and Lévy index, which is (similar to the present problem) “responsible” for the degree of disorder. All other microscopic characteristics of the defect ensemble were shown to be irrelevant. This means that the theoretical method of^41^ is similar to ours in the sense that it gives the effective averaging over disorder without any additional assumptions about its (disorder) microscopic details.

In our problem, one of the physical effects of the interplay of Coulomb interaction screening and disorder is the possibility for exciton to collapse at weak screening or to break up at strong one. As stated above, these effects cannot be realized in ordered substance, i.e. at $\mu =2$. The next important effect is energy levels crossing^52^, appearing also due to the joint action of Coulomb screening and disorder. The latter effect is related to the levels repulsion and their non-Poissonian statistics, inherent in quantum chaotic systems. This might point to the possible chaotic features of exciton motion in the above 2D dielectric structures, especially in the presence of spin-orbit interaction^63^. The point is that recently such chaotic features have been found in the excitonic spectra of 2D structures with unscreened Coulomb interaction, but with the inclusion of Rashba spin–orbit interaction^64,65^. In context of free electrons and holes, the role of latter interaction in 2D electron gas confined in GaAS quantum well had been studied in^66^. This suggests a generalization of the present problem. Namely, the spin–orbit interaction term can be added to the corresponding fractional Schrödinger equation. In this case, the solution will be more sophisticated as the wave function will be spinor now^65^ although the problem can possibly be solved in the momentum space similar to the present consideration. Such a problem turns out to be extremely important for the above classes of substances^67^ where chaos can even disrupt the functionality of corresponding optoelectronic and/or photovoltaic devices. It has been shown^65^ that the description utilizing the “ordinary” (i.e. that both with conventional Laplacian and unscreened Coulomb interaction) hydrogenic problem does not show strong quantum chaotic features like non-Poissonian energy level statistics, see, e.g.,^68^. This may be related to the fact that proper description of such features is possible only within excitonic models, containing fractional Laplacians and screened Coulomb interaction, inherent in the majority of semiconductors. Similar to the above “chaotic models”, which stem from averaging over different microscopic disorder realizations, our 2D fractional hydrogenic model is limited to the distances of about 10 lattice constants i.e. around 40-50 Å. This is because at such distances, the description in terms of material constants (like permittivities $\varepsilon$) is possible and on the other hand, the effective averaging over disorder have been performed resulting in the fractional Laplacian introduction in the Schrödinger equation.

The described effects can play an important role in the relaxation of the energies of electron and hole, bound in an exciton. In a disordered 2D substance, instead of a process with well-defined time dependence, the energy relaxation from a highly excited to the ground state may become chaotic. This can obviously hinder useful photovoltaic processes (like conversion of solar energy to electric one) since the disorder may reduce the controllability of the photovoltaic performance.

There is a significant corps of articles, devoted to optical properties of excitons in geometrically confined environments like quantum wells (2D)^69,70^ and quantum wires (1D)^71,72^ with the disorder, introduced either by fluctuations of the quantum well width^70^ or random potential^69^. These kinds of disorder had been treated within the formalism, based on ordinary Schrödinger equation with subsequent averaging over disorder^69^, see Ref.^73^ for details of averaging procedure in^70^. Moreover, the randomness of the potential in Ref.^69^ was of the white noise type (see expression (12) of^69^) with ordinary (i.e. non-fractional) Gaussian fluctuations. The main qualitative features of the above considerations coincide with our results. Namely, the more disorder is in the system, the “more localized” in ${\mathbf{r}}$ space the exciton wave function is. Although here we study the spectrum of the 2D fractional hydrogenic problem, this study has been fulfilled in the environment with highly non-Gaussian fluctuations. The results obtained here will in the future be used to calculate the observable characteristics (like spectra of absorption, radiative lifetimes, etc) of the excitons based on our model of fractional Schrödinger equation.

An interesting generalization of our 2D fractional hydrogenic problem is consideration of exciton-phonon bound states—so-called excitonic polarons, see Ref.^74^ and references therein. As the coupling between an electron and a hole in such polaron occurs via flexural phonon modes, which are spread between several (usually two) TMD monolayers, this problem may be considered an intermediate between 2D and 3D situations. Once more, the introduction of fractional Laplacian to the corresponding Schrödinger equation (and eventually to the equations, governing the properties of phonons) can be important to consider the effects of disorder, which in such bilayer structure may change the optical absorption and emission spectra drastically, see Ref.^74^ and references therein.

One more generalization of the problem considered is to address the 3D fractional quantum-mechanical hydrogenic problem with screened Coulomb interaction. This problem is important for Rydberg excitons (described by the quantum mechanical Kepler problem, see, e.g.^75^) in amorphous (i.e. highly disordered) bulk semiconductors. This additional dimension may generate certain subtleties (for instance the system may be prone to chaos, see^64,65^) as compared to present 2D screening, especially in the presence of Rashba spin-orbit coupling^7^.

## Methods

The details of our theoretical methodology and those of working with fractional derivatives and fractional Laplacians, in particular, have been described in the “Theoretical approach” section. The numerical solutions of spectral problems for integral equations have been conducted using the commercial *Mathematica* software package. We perform the numerical solutions for each specific *m* (orbital quantum number). Note that numerical calculations have been performed in dimensionless variables (12).
